# A novel COVID-19 program, delivering vaccines throughout rural and remote Australia

**DOI:** 10.3389/fpubh.2023.1019536

**Published:** 2023-07-17

**Authors:** Fergus W. Gardiner, Zoe Schofield, Miranda Hendry, Kate Jones, Mandy Smallacombe, Mardi Steere, Jenny Beach, MaryBeth MacIsaac, Randall Greenberg, Candice Crawford, Melanie Trivett, Judah Morris, Breeanna Spring, Frank Quinlan, Leonid Churilov, Kris Rallah-Baker, Elli Gardiner, John O’Donnell

**Affiliations:** ^1^Royal Flying Doctor Service of Australia, Canberra, ACT, Australia; ^2^The Rural Clinical School of Western Australia, The University of Western Australia, Perth, WA, Australia; ^3^CDU Menzies School of Medicine, Darwin, NT, Australia; ^4^Molly Wardagua Research Centre, Faculty of Health, Charles Darwin University, Darwin, NT, Australia; ^5^Melbourne Brain Centre at the Royal Melbourne Hospital, The University of Melbourne, Melbourne, VIC, Australia

**Keywords:** COVID-19, remote, vaccine, vaccination, community, engagement, pandemic

## Abstract

**Background:**

The Royal Flying Doctor Service of Australia (RFDS) established a unique SARS-CoV-2 vaccination program for vaccinating Australians that live in rural and remote areas. This paper describes the preparation and response phases of the RFDS response.

**Methods:**

This study includes vaccinations conducted by the RFDS from 01 January 2021 until 31 December 2021 when vaccines were mandatory for work and social activities. Prior to each clinic, we conducted community consultation to determine site requirements, patient characteristics, expected vaccination numbers, and community transmission rates.

**Findings:**

Ninety-five organizations requested support. The majority (*n* = 60; 63.2%) came from Aboriginal Community Controlled Health Organizations. Following consultation, 360 communities were approved for support. Actual vaccinations exceeded expectations (*n* = 70,827 vs. 49,407), with a concordance correlation coefficient of 0.88 (95% CI, 0.83, 0.93). Areas that reported healthcare workforce shortages during the preparation phase had the highest population proportion difference between expected and actual vaccinations. Areas that reported high vaccine hesitancy during the preparation phase had fewer than expected vaccines. There was a noticeable increase in vaccination rates in line with community outbreaks and positive polymerase chain reaction cases [*r* (41) = 0.35, *p* = 0.021]. Engagement with community leaders prior to clinic deployment was essential to provide a tailored response based on community expectations.

## 1. Introduction

By 22 February 2022, SARS-CoV-2 (COVID-19) pandemic had infected over 422 million people and directly caused 5.8 million deaths (1). The COVID-19 Delta Variant B.1.617.2 was the prominent strain from May 2021 until December 2021. This strain had a basic reproductive rate (R_0_) between 5 and 8 making it 60% more transmissible than the Alpha variant (R_0_ 2.2; 95% CI, 1.4–3.9) (2, 3). Since late December 2021, the Omicron (BA·1 and BA·2) strain has become the prominent strain with a potential R_0_ as high as 10, with cases reported as doubling every 2–3 days (4).

Public health measures differed geographically to achieve control of transmission. The Australian national response involved public health test-trace-isolate-quarantine responses and public health and social measures (5). Because of the high R_0_ associated with the Delta and Omicron variant, the World Health Organisation (WHO) advised that public health, social measures and vaccination are essential in reducing transmissibility and hospitalization (1). Based on modeling conducted by the Peter Doherty Institute for Infection and Immunity, strict lockdowns were required to manage outbreaks until 70–95% of people aged 16 years and over had received 2 doses of the COVID-19 vaccine (6).

Rural and remote populations are at greater risk of severe respiratory disease than their major city counterparts. They have higher rates of chronic disease, and less access to primary healthcare services and emergency departments (6, 7). Aboriginal and Torres Strait Islander Peoples account for 15% of the population in remote areas and 49% of the population in very remote areas (8). There was specific concern about the susceptibility of these populations to COVID-19 due to larger family sizes and increased household mixing that could make them especially susceptible to the Omicron BA.2 variant which has a higher potential R0 (6). Vaccination rates over 70% were the target but in these populations 95–100% was speculated to be required to minimize the spread and reduce severe outcomes (6, 7).

Operation COVID Shield, the national vaccine rollout, was an operational task-force to ensure all workers had access to vaccination before the September 2021 deadline. This effort was mostly carried out by road-mobile vaccination crews traveling to hospitals and aged care facilities, and then through General Practice-led respiratory clinics. Almost 90% of Australians had access to these services. This is because the majority of Australians live in major cities (69%) and regional areas (20%). However, other strategies were required to vaccinate the remaining 11%, which was 3.86 million people that live in remote and very remote areas.

The RFDS had been providing aeromedical responses to COVID-19 since February 2020, when the first aeromedical retrieval for a COVID-19 patient occurred from Darwin. Since then, the RFDS has conducted over 7,500 COVID-19 retrievals by road and air ambulance (9). The RFDS has existing relationships with remote communities through aeromedical retrieval, and more recently, through the mobile primary health care services we provide to rural and remote communities. We were, therefore, in a unique position to provide vaccination clinics to the most vulnerable populations living in remote areas. We were engaged by the Australian Federal Government to assist in “Operation COVID Shield,” and tasked in early 2021 to conduct vaccinations in rural and remote Australia (10).

This paper outlines the RFDS COVID-19 vaccination program preparation and response phases, highlighting key factors that were integral to the vaccination program.

## 2. Methods

### 2.1. Setting

Australia covers an area of 7.69 million square kilometers, making it the sixth largest country by total area (11). However, Australia has a small population (*n* = 25.74 million) with most of the Australian population living in major cities (*n* = 18.5 million, 72.0%), or inner-regional areas (*n* = 4.6 million, 18.0%) areas (12). The remaining population live in outer-regional (*n* = 2.1 million, 8.0%), remote (*n* = 0.3 million,1.1%), and very remote areas (*n* = 0.2 million, 0.8%) (12).

The RFDS provided a vaccine service to remote and very remote areas and delivered vaccines to non-RFDS clinics in outer regional, remote and very remote areas. This study includes data for vaccines given to remote and very remote Australian residents, as defined by the Australian Statistical Geography Standard (ASGS) (13). Remote and very remote Australia has low population concentrations distributed over vast distances with limited or no service provision, including healthcare, for hundreds of kilometers (14, 15).

Major city areas and inner regional areas, were excluded from the analysis, as these non-rural locations were not a focus of the RFDS COVID-19 response as outlined in the “National COVID Vaccine Campaign Plan” (10).

This study involves COVID-19 vaccination activities carried-out by the RFDS. We have not included vaccination efforts provided by external service providers; specifically, permanent primary healthcare services. The RFDS is the largest aeromedical service in the world, providing primary evacuations and inter-hospital transfers by road and air. Besides the aeromedical service, the RFDS provides extensive primary healthcare outreach services to areas without traditional service provision, operated in a hub and spoke model, including, although not limited to, nursing, general practitioners, oral health, and mental health services. In the 2019/20 financial year the RFDS conducted 37,666 aeromedical and 62,895 road ambulance retrievals, and over 320,000 primary healthcare patient episodes of care (9).

### 2.2. Royal flying doctor service COVID-19 vaccination clinics

The RFDS COVID-19 program is consistent with an Alliance Governance Framework (16). Alliance Governance Frameworks within healthcare are designed to improve patient care in a systematic and accountable manner, while reducing overall costs by encouraging providers to pool funds and share resources. During an alliance governance contractual arrangement, all organizations are equal partners, with all parties sharing the risks and responsibilities (16).

Because of the quickly changing COVID-19 situation, the RFDS and the Commonwealth Government entered a formal agreement quickly and in good faith to assist in the provision of an aeromedical and vaccination response. While the agreement specified essential reporting and contractual obligations, such as data reporting, the partnership in practice included the following activities:

1. High acuity aeromedical retrieval services (primary evacuations).
2. Low acuity aeromedical retrieval and patient transport services (early and secondary evacuations).
3. Mobile General Practitioner (GP) Respiratory Clinic Services.
4. Personal Protective Equipment (PPE) delivery services.
5. COVID-19 vaccination services, using either road or aeromedical based transport to regions.

The RFDS vaccination program reported to the Commonwealth Department of Health, Indigenous and Remote COVID-19 Governance, Engagement and Response Branch. Although not limited to vaccinating Aboriginal and Torres Strait Islander populations, we were tasked to service remote and very remote Indigenous communities. The Australian Government was rolling out the vaccine in stages to at risk populations and then by age. Due to the logistics involved in serving remote and very remote communities our remit was to focus on the whole of population vaccination of those aged 16 years or older using the Pfizer-BioNTech COVID-19 (Pfizer). The first approved vaccination clinic (16 years or older) was conducted on 20 January 2021. RFDS began delivering vaccines to 5–11-year-olds from 03 December 2021. Child vaccines have not been included in this analysis.

### 2.3. Vaccination assistance service

The RFDS in partnership with the Commonwealth government and community health services including National Aboriginal Community Controlled Health Organisations developed the Vaccination Assistance Service (VAS) form. Communities and health services requiring RFDS vaccine support had to complete the form, which was then sent to the Commonwealth for approval. The Commonwealth Government provided the VAS form to State and Territory Departments of Health and National Aboriginal Community Controlled Health Organisation. The RFDS communicated the VAS form via its health networks.

The VAS form requested information to help plan and deliver vaccines. It requested information such as patient numbers and population demographics, community and clinical support, and special requirements for communities (Table 1). During each vaccination clinic, we recorded patient data on the Australian Immunisation Register (AIR) as mandated by the AIR Act 2021. In addition to entering information onto AIR each clinic recorded patient details into RFDS clinical software and recorded vaccine numbers for reporting to the commonwealth.

**Table 1:** Information requested on the VAS form to support service planning and delivery of COVID-19 vaccines.

Requested by
Location
Population/audience
Population 50 and over, incl. Indigenous population (%) if known
Population 16–49, incl. Indigenous population (%) if known
Jurisdictional support
Community consultation
ACCHS/ NACCHO affiliate support
Vaccine doses available
Vaccine administrator credentials
Logistics
Dates for planned vaccination and priority dates for requested workforce support
Duration
Current resources available within jurisdiction
Commonwealth resources requested (in addition to current resources available)
Information needed from Commonwealth to assist with this request
Freezers/storage
Other resources/information
Communications/community preparedness
Sensitivities/considerations
Specific community requests (e.g., wear civilian clothing)
Vaccine lead agency
Jurisdictional delegate approved

### 2.4. Statistical analysis

RFDS vaccination clinic categorical variables, such as vaccination numbers and population rates, were summarized as counts and proportions. Comparisons between the patient genders receiving a vaccination was made by estimating 95% confidence intervals for means and proportions of interest for the samples and examining whether the population values belonged within those confidence intervals. The population value outside of the sample 95% confidence interval can be interpreted as indicative of a statistically significant difference between the samples at the significance threshold of 0.05.

We conducted Lin’s concordance correlation coefficient to compare the proportion of patients expected (based on community consultation) relative to population census information at the vaccination clinics prior to RFDS deployment and then the actual RFDS vaccination population proportion relative to population census figures. We conducted a Chi-Square analysis to determine whether expected and actual vaccination proportions differed by geographical area. The proportion dominator used was the total population per town/community less 23% to account for the ineligible population which, at this time, were people under 16 years old (12).

To determine whether vaccination rates were higher in areas with high COVID-19 transmission, we used data directly obtained from the National Notifiable Disease Surveillance System (NNDSS). To reflect the RFDS vaccination clinic dates, the NNDSS data included positive COVID-19 polymerase chain reaction (PCR) results by geographical regions from 01 January 2021 until 31 December 2021. The study period was before Rapid Antigen Tests (RATs) were available in Australia. Using the Australian Statistical Geography Standard, data is reported based on the Statistical Area Level 3 (SA3). SA3 regions provide a regional breakdown of Australia and have a population between 30,000 and 130,000 people. Using SA3 areas allows the RFDS to compare similar areas for vaccine delivery. We then conducted a Pearson’s correlation coefficient to determine whether there was a relationship between the proportion of positive cases reflective of population numbers and the proportion of the population by geographical region in which the RFDS provided doses. The proportion dominator used was the total population per geographical region (SA3), with population data obtained from the Australian Bureau of Statistics (12). Statistical analyses were performed using the statistical software package R version 3.5.1.

### 2.5. Ethics approval

This project was deemed a low-risk quality assurance project by the RFDS Clinical and Health Services Research Committee (CHSRC), on 12 June 2020. All methods were carried out under Australian HREC guidelines and regulations.

### 2.6. Role of the funding source

The RFDS COVID-19 response was funded and supported by the Commonwealth Government of Australia, with the RFDS functioning as a member of the Department of Health and Department of Defence “*Operation Shield*.” The Commonwealth Department of Health was a key stakeholder in the RFDS COVID-19 Vaccination response, as detailed in the below section.

## 3. Results

### 3.1. Preparation phase

**Figure 1:**
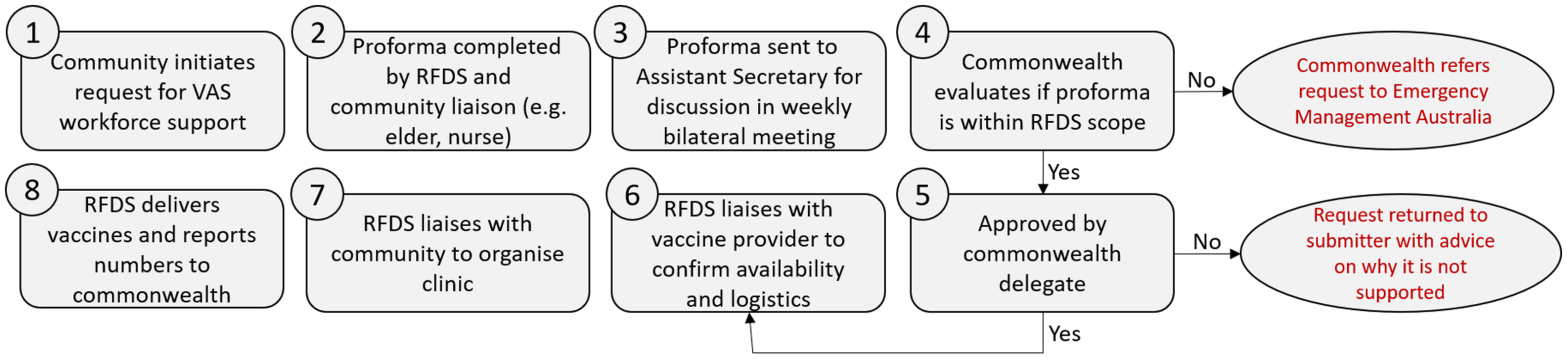
Workflow showing how vaccine assistance service (VAS) requests for RFDS was processed and approved or rejected through the Commonwealth.

In practice, this phase resulted in community leaders and representatives throughout Australia completing a VAS request form (Table 1). This VAS form was completed in coordination with state and territory-based RFDS personnel and the relevant Health Organisation and approved by the Commonwealth (Figure 1). It was then progressed through the VAS governance process for final approval by the Commonwealth. This strategy helped to ensure that the clinics were developed and completed in a community-informed manner.

Throughout the study period, the RFDS was approached by 95 separate bodies, requesting vaccination support via the VAS process. The majority (*n* = 60; 63.2%) of the requests came from Aboriginal Community Controlled Health Organisations (ACCHOs), such as Miwatji Health and Nganampa Health Service, with the rest coming from State or Commonwealth agencies (*n* = 35; 36.8%), such as state/territory Departments of Health and Hospital Health Networks. This resulted in 102 separate VAS forms being submitted and approved. Working with communities helped to identify when a community would not be eligible and provide guidance on alternative vaccine access. Fifty-five submitted VAS forms were rejected because the requests came from communities in inner-regional or major city areas. This was outside of the RFDS vaccination remit which, was to focus on remote and very remote Australia.

Following initial VAS form submission, the relevant RFDS State Officer (SO) (State/Territory RFDS COVID-19 lead) and the RFDS Federation Officer (FO) (National COVID-19 lead), conducted the following suitability assessment:

▪ Stakeholder engagement—including community consultation and yarning—an important process within Aboriginal and Torres Strait Islander culture to build respectful relationships.
▪ Modification of VAS form depending on community needs, in consultation with local health leaders and RFDS deployment leader (RFDS Senior Vaccination Officer).
▪ SO, RFDS Senior Vaccination Officer, and community representatives developed a deployment strategy and timelines.
▪ RFDS FO and SO discussed with the Commonwealth at weekly bilateral meetings.
▪ RFDS FO and the Commonwealth First Assistant Secretary (FAS) evaluated for deployment approval.
▪ RFDS SO and RFDS Senior Vaccination Officer conducts community consultations and engagement prior to deployment, including although not limited to vaccination information sessions and advertising.

Throughout the study period, this flexible process resulted in 360 separate community areas being approved for whole of community vaccination deployments some of which were in the most remote areas of Australia.

### 3.2. Stakeholder engagement

Stakeholder engagement was essential for the vaccination rollout. Initially the RFDS attempted to provide vaccines to communities on an *ad-hoc* basis. This was unsuccessful in Indigenous and non-Indigenous communities, especially in communities outside of the regular RFDS service footprint. A lack of trust combined with vaccine hesitancy and misinformation made the rollout very challenging and after canceling clinics we needed to take a different approach. The teams on the ground led the approach, but a common theme was to engage with communities prior to vaccination and empower them to request the vaccine assistance service (Figure 1, Step 1). The RFDS teams engaged with local mayors, councils and disaster management groups to identify the needs of different communities. The teams attended community events; organized events in communities to provide information on the virus and the vaccine; and engaged in Yarning with Indigenous communities and their elders. Yarning is an extremely important tool in Indigenous communities to build respectful relationships and trust (17). RFDS teams met with community leaders prior to clinic deployment and provided tailored responses based on community expectations (Figure 1, Step 7). RFDS teams undertook specific cultural awareness training to ensure they understood cultural sensitives before arriving.

A concern shared by many stakeholders was RFDS spreading the COVID virus into their community. Prior to Rapid Antigen Testing, crews took their temperatures daily and always wore full PPE in community. In Kowanyama, RFDS teams agreed to extend their visits from the usual 3 days per week to 3 weeks to deliver primary care, build trust with the community through continuity of care and provide a familiar face to ask questions about the vaccine to combat mis-information and reduce vaccine hesitancy.

To vaccinate people in remote communities we had to adapt to the local environment as the usual clinic environment was not available. Vaccine clinics had to be easily accessible and where people felt comfortable. In the logistical planning phase (Figure 1, Step 8) some unusual locations were identified. These included pubs, shearing sheds, marquees provided by local councils, outside grocery stores and under trees. We visited homes, cattle stations and mine sites. In Tasmania, two former public transport buses became vaccine clinics on wheels delivering over 1,300 vaccines to small townships. In Galiwin’ku RFDS crews delivered vaccines in a rugged four-wheel drive vehicle that became affectionately known as the “Vaxi Taxi.”

There were many challenges around logistics, navigating diverse stakeholders, vaccine hesitancy, language and cultural barriers, outbreaks, and available transport and accommodation. This was a huge learning curve making us adaptable and creative in the delivery of vaccines and education. We employed Aboriginal Liaison Officers from within community who were invaluable in community engagement and logistical support. Local community members helped to translate public health messages.

In some Indigenous communities the red in our uniforms was seen as disrespectful and the navy blue was too similar to the police. In these locations, uniforms were redesigned so they were culturally appropriate and clearly identified us as health care providers.

### 3.3. Response phase

**Figure 2:**
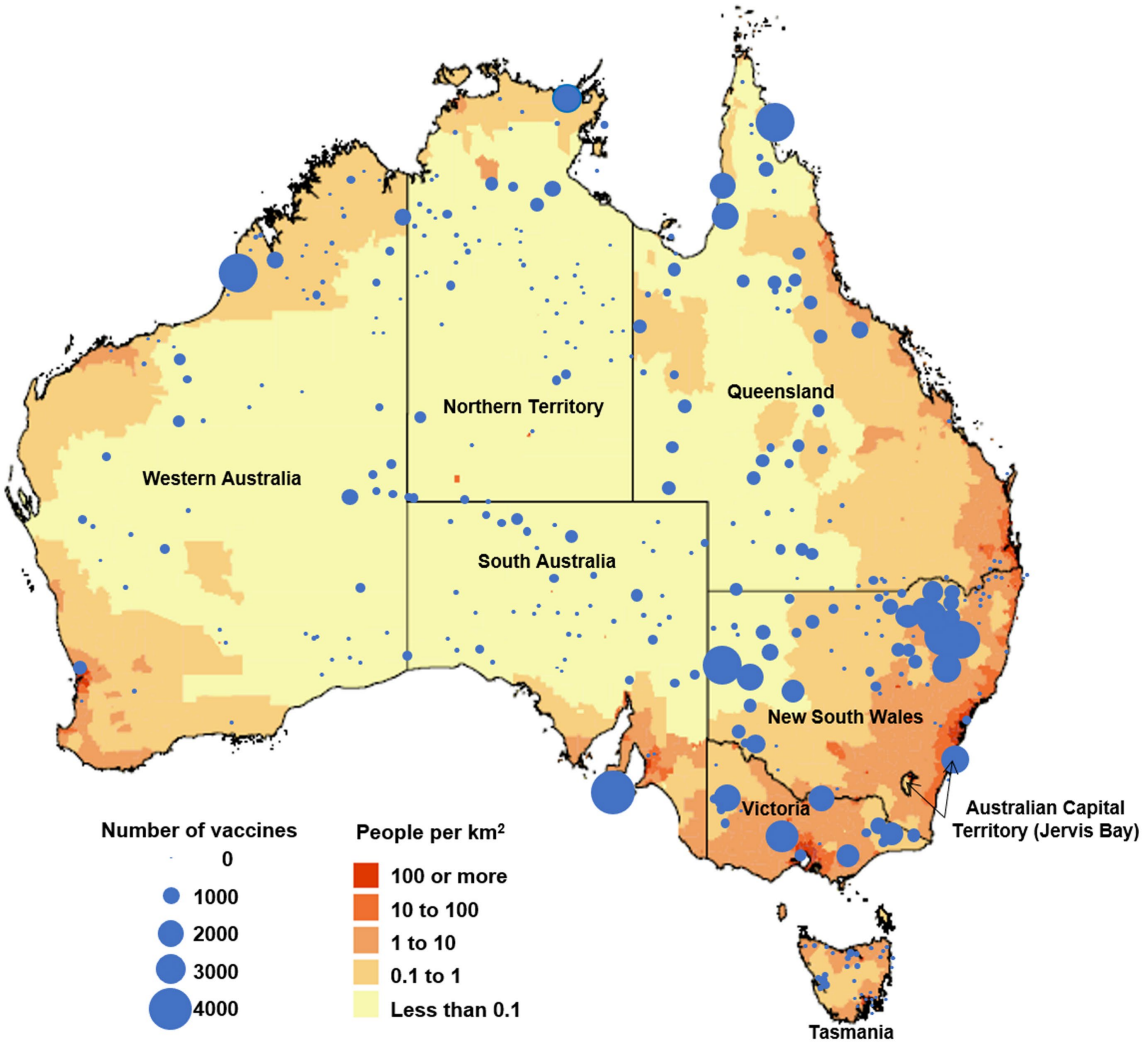
Map of Australia showing population density per square kilometer (adapted from Australian Bureau of Statistics) (12) and The Royal Flying Doctor Service COVID-19 vaccination clinic locations and number of vaccines given between 20 January 2021 and 31 December 2021.

Following the preparation phase, the RFDS conducted clinics throughout Australia, resulting in 70,827 vaccinations (dose 1 and 2) given from the 20 January 2021 to the 31 December 2021 across remote and very remote populations (Figure 2). The Australian-wide RFDS vaccination clinic median patient age was 51 years (mean = 49.0 years). A third (33.0%) of people vaccinated identified as Aboriginal or Torres Strait Islander, even though only 3.3% of the population identifies as Aboriginal or Torres Strait Islander.

In addition to providing vaccinations directly, the RFDS also provided 19,670 Pfizer vials (6 doses per vial using low dead space needles) by aircraft and road ambulance to other service providers.

Reflecting on the higher rates of COVID-19 in New South Wales and the heightened threat of community transmission, we carried out the most vaccines in New South Wales during this period (*n* = 29,589). Moree and Narrabri with an eligible population of approximately 15,100 received the highest numbers of vaccinations conducted by the RFDS (*n* = 8,264, 54.5% of the local population) followed by Gunnedah (*n* = 7,908, 78.5% of the local population), Broken Hill and Far West (*n* = 7,284, 21.1% of the local population), Far North (*n* = 6,712, 32.6% of the local population), and the Kimberley (*n* = 4,803, 17.8%).

**Figure 3:**
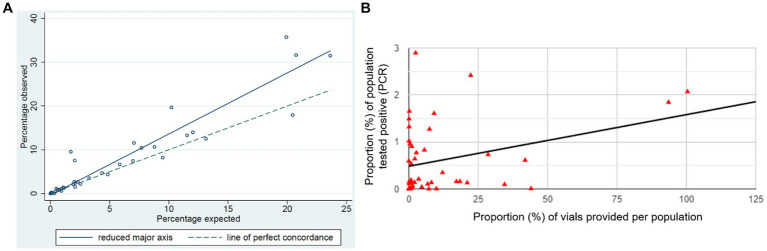
Proportion of the population **(A)** expected to be vaccinated, as per VAS request form compared to actual vaccinations and **(B)** correlation between vaccination rates and positive (PCR) COVID results.

Actual vaccinations were significantly higher than predicted (Table 2), with 49,407 expected and 70,827 vaccinated with a Lin’s concordance correlation coefficient of 0.88 (95%CI: 0.83, 0.93). When comparing the expected and actual vaccination rates by population, we found that all the following areas had significantly (*p* < 0.05) higher actual vaccination rates than expected (Figure 3A). Moree–Narrabri (23.6% vs. 31.5%), Broken Hill and Far West (20.0% vs. 35.7%), Beswick (20.7% vs. 31.6%), Far North (10.2% vs. 19.7%), and East Arnhem (7.1% vs. 11.6%). During the preparation phase, these areas identified workforce shortages, service inaccessibility, and the rates of potential community COVID-19 transmission, as a major motivator to request RFDS. The areas of Outback—South (20.5% vs. 17.9%), Barkly (13.1% vs. 12.5%), Katherine (9.4% vs. 8.2%), and Goldfields (4.85% vs. 4.4%) all had significantly (*p* < 0.05) fewer vaccinations than expected. During the preparation phase, these areas identified workforce shortages and service inaccessibility. These areas also had high population vaccine hesitancy or deliberation.

**Table 2:** Vaccines given by RFDS-led respiratory clinics by state/territory.

Australian state/territory	Total expected RFDS vaccinations based on the VAS consultation process	Vaccinations administered by the RFDS (dose 1 + 2)	Proportion difference (expected vs. actual)	Confirmed COVID-19 cases (PCR) reported in the RFDS operating area
Australian Capital Territory (Jervis Bay area)	542	1,160	2.1	0
New South Wales	17,163	29,589	1.7	5,980
Northern Territory	4,711	5,215	1.2	417
Queensland	9,169	11,875	1.2	206
South Australia	3,632	7,344	1.8	1,556
Tasmania	1790	2031	1.2	324
Victoria	3,250	3,896	1.1	4,052
Western Australia	9,150	9,717	1.2	29
Total	49,407	70,827	1.4	12,564

Between 01 January and 31 December 2021 there were 12,564 positive PCR pathology results in the RFDS operating area, with Shepparton (*n* = 1,897; 15.1%), Dubbo (*n* = 1,487; 11.8%), Tamworth–Gunnedah (*n* = 1,054; 8.4%), and Shoalhaven (*n* = 1,052; 8.4%) having the highest positive PCR rates. Results indicated there was a positive relationship between the proportion of the population who had a positive PCR result and the proportion of population vaccinations provided [*r*(41) = 0.35, *p* = 0.021] by geographical region (Figure 3B). Showing that vaccinations increased in line with community COVID-19 transmission.

Across Australia, 64,382,557 vaccine doses were administered between February 2021 and December 2021. The RFDS’s contribution seems small in comparison. However, our vaccination remit was to provide vaccines to remote and very remote communities that could not access vaccines through their primary health network. The population in remote and very remote areas is approximately 500,000 people. Of the eligible population (approx. *n* = 385,000), we provided 70,827 vaccines directly to almost 20% of the population *via* our RFDS vaccination teams. When including vial deliveries (118,020 doses provided) we were directly involved in the provision of 188,847 vaccinations. This covered nearly 38% of the remote and very remote population. The majority of our vaccinations went to areas with a high proportion of Aboriginal and/or Torres Strait Islander peoples. Without our efforts, these communities would have struggled to access vaccines. This would have put these communities at extreme risk of respiratory illness, hospitalization and death, as borders reopened and travel commenced. Where vaccination rates were lower, PCR positive COVID cases were higher highlighting the importance and benefit of vaccines prior to outbreak.

## 4. Discussion

High vaccination numbers are essential to minimize and prevent community COVID-19 transmission. A key element of the RFDS COVID-19 response, was partnering with communities *via* the VAS alliance framework to ensure historically marginalized groups were given priority in the deployment of community informed mobile vaccination and pop-up clinics. The alliance framework gave the RFDS flexibility to collaborate with local community members and specifically adapt each clinic deployment based on community need.

Interestingly, community uptake of vaccination was significantly higher than estimated in the VAS consultation process. This was especially true in NSW, where COVID-19 transmission was highest during the study period. During the preparation phase, reasons cited for barriers to vaccination were workforce limitations and service inaccessibility. A recent study found intention to be vaccinated by rural people was influenced by limited vaccine appointment availability, or their local clinic not having vaccines available (18). Travel times for rural and remote people to their nearest inner-regional respiratory clinic hub (vaccination and testing) has been identified as a barrier to vaccination (18, 19). To combat these barriers, the RFDS engaged early and often with communities and provided locally available vaccine clinics with clear messaging about availability. This in combination with the RFDS being a respected organization (20), could have resulted in the increased number of people traveling to RFDS clinics to receive vaccination.

Several communities had lower than expected vaccination rates. We did not report vaccines provided by external service providers who could have serviced those community members, as data sharing between services was limited during the height of the pandemic. Another possibility for the lower numbers, as identified in the consultation phase, is a high level of vaccine hesitancy. Vaccine hesitancy is complex and a detailed analysis is beyond this research This is an international issue and interestingly, the literature indicates that the barriers and solutions to COVID-19 vaccination efforts were similar in the United States of America (21), the United Kingdom (22), and Guinea (23). The World Health Organisation has identified three core barriers to vaccine uptake which are complacency, confidence, and convenience. These will be discussed briefly in the context of our experience vaccinating rural and remote communities in Australia.

### 4.1. Complacency

One of the factors influencing a decision to get vaccinated is a belief that you are at high risk of getting COVID-19 and/or will suffer severe complications (24). Early in the COVID-19 pandemic, many Australians, especially those living in rural and remote Australia, enjoyed a COVID-free environment or had few cases to cause alarm. This could have increased denial about the seriousness of COVID-19 thus increasing the chance of catching COVID-19. Our data reflects a significant increase in vaccination rates over expected numbers in communities that had higher numbers of confirmed cases.

### 4.2. Confidence

Some groups reported to be unsure or unwilling to undergo vaccination. This is related to concerns on the long-term side effects and adverse reactions of the vaccine (25), and skepticism about its efficacy (26). This has been compounded by COVID-19 vaccine misinformation, especially on social media (27). Our findings show an increase in vaccination appointment cancelations, especially in Far West New South Wales and the Cape regions of Queensland and Northern Territory, due to negative social media posts.

Misperceptions about COVID-19 have been linked to lower health literacy and less knowledge about vaccines (28). Lower socioeconomic status is linked to poorer health literacy (28). People living in rural areas have lower socioeconomic status compared to urban areas. This contributes to the lower levels of health literacy in rural populations in Australia (36% compared to 42% in metropolitan areas) (29). Health literacy levels are lower in migrant, refugee and indigenous groups compared to the general Australian population (79% compared to 54%) (29). A consequence of Australian Government settlement policies means many of these populations live in rural and remote Australia.

Vaccine hesitancy is also caused by a deep-rooted and ongoing mistrust of the medical system and an under representation of suitably diverse populations in clinical trials (30, 31). Our observations in the field confirmed that vaccine hesitancy, especially in Indigenous populations, was associated with mistrust rooted in intergenerational trauma associated with colonization. To improve vaccine uptake RFDS staff engaged with individual communities and built respectful relationships and trust through yarning and engagement with community leaders and elders. This led to over one third of our vaccines going to people that identified as Aboriginal and/or Torres Strait Islander. The importance of community engagement was one of the most important lessons learnt by RFDS and laid a foundation of trust for future service provision. As COVID-19 spread across Australia, and people’s fears of catching COVID-19 increased, RFDS were invited to provide vaccine support by communities. Being ready to support these communities and building community relationships meant that vaccination rates in some of Australia’s most vulnerable communities increased (24).

### 4.3. Convenience

General Practice (GP) and nursing clinics play a central role in disease detection, notification, treatment and prevention through health promotion and vaccination (32). However, the COVID-19 pandemic highlighted underlying problems with rural and remote healthcare provision. It showed that many communities have limited access to basic essential services such as GP and nursing services (33). This meant significant short-term surging of the workforce was necessary to fill the gaps. This in part, mitigated the impact on permanent services and provided vaccination services. However, this is not a suitable long-term solution. It is clear that a robust health care workforce is required in rural and remote communities to improve health literacy, and trust within health services in preparation for future pandemics and other public health threats (34).

### 4.4. Key lessons learnt for future pandemics/responses

The RFDS is a prominent provider of aeromedical and primary health care in rural and remote communities across Australia. We were therefore well positioned to respond during the COVID-19 pandemic. This effort was not without challenges and we identified key lessons that will enable us to respond to future pandemics and other health care issues more effectively.

#### 4.4.1. Maldistributed health workforce

There is an undersupply of health workforce in rural Australia. This meant that local health services were unable to provide a vaccination service or travel to remote communities to do it. As such, surge workforce models need to be embedded into future response planning, factoring workforce fatigue, training, and the need to maintain routine patient care.

#### 4.4.2. Improving supply chains

We spent a considerable amount of time establishing reliable supply chains for vaccines, vaccine consumables and personal protective equipment within geographically isolated environments. Rural and remote areas need early provision of essential resources. Many rural communities in Australia have high rates of chronic disease, especially type 2 diabetes and were at high risk of COVID

#### 4.4.3. Community consultation and engagement

This was, indubitably, the main lesson we took from this process. We realized that our community engagement differed across Australia and different approaches were required to build relationships. Individual community consultation prior to vaccine roll-out was essential to the success of our vaccine efforts. We will continue to develop and establish relationships at the community level so we are better placed to deliver health care services to remote and very remote communities. The RFDS is aligning its service delivery with the Comprehensive Care Standard. This integrates patient care processes to identify individual needs and the needs of smaller populations. This focuses on community engagement and co-design to ensure services are suitable for the needs of unique communities.

#### 4.4.4. Communication barriers

We need to improve communication to ensure that we are communicating effectively and providing trustable and respected information. The obvious barrier is communicating in different languages. To address this we need local language communique that include local totems and images that are translatable, and translators in communities. We also need to address communication barriers between clinicians and patients. To achieve this we need to improve cultural understanding to improve how clinical information is related to the patient.

#### 4.4.5. Consistent population vaccination data and transmission rates

Future planning approaches need to systemize data entry and reporting across Australian states and territories to avoid inconsistencies and delays. This will enable targeted response measures in a rapidly changing environment based on the needs of specific areas and/or populations.

Through lessons learned early about stakeholder engagement and understanding of the culturally diverse populations in Australia, RFDS was able to build trust within communities and health care providers. This enabled a successful collaborative venture resulting in over 70,000 vaccines being delivered in less than a year. The total number of vaccines given exceeded the expected number by 50%.

### 4.5. Limitations

The main limitation associated with this study, was that it only included data from a single vaccinating service, however this limitation was reduced in that many of the deployed clinics were the only vaccinating service in the geographical area.

A limitation could be associated with using actual vaccination numbers as an indicator of program success. While RFDS vaccination numbers were more than expected as indicated by the concordance correlation coefficient results, there were some areas with low overall (RFDS and non-RFDS) vaccination uptake. Specifically, overall vaccination uptake was also likely influenced by external factors, such as government education interventions. However, this limitation was reduced by receiving expected vaccination numbers directly prior to clinic deployment during the community consultation phase. This enabled us to estimate the number of vaccines required for each deployment.

## 5. Research in context

### 5.1. Evidence before this study

Published research on SARS-CoV-2 (COVID-19) has focused on transmissibility and effectiveness of public health measures. There is little information on implementing the national vaccination program throughout isolated areas of Australia.

### 5.2. Added value of this study

Australia’s national vaccination program, Operation COVID Shield, implemented vaccines across Australia. This was to be rolled out through vaccine teams and general practice. In rural and remote Australia where access to health care facilities is limited the strategy fell short. The Royal Flying Doctor Service (RFDS) were contracted to support vaccination clinics in rural and remote areas to fill this gap, *via* a surge workforce. Ninety-five independent organizations requested vaccination support *via* the vaccination assistance service (VAS) framework. The RFDS administered more vaccinations than expected. This demonstrates the barrier to accessing health care and the need and effectiveness of community consultation and adapting clinics to meet the needs of the community.

### 5.3. Implications of all the available evidence

Vaccination responses to a pandemic need to be adaptive and provide appropriate interventions reflective of community need.

## Data availability statement

The raw data supporting the conclusions of this article will be made available by the authors, without undue reservation.

## Ethics statement

The studies involving human participants were reviewed and approved by ACT health, Centre for Health and Medical research, ACT Health Directorate. The patients/participants provided their written informed consent to participate in this study.

## Author contributions

FG: conceptualization. FG, MH, and LC: data curation. FG and MH: formal analysis. FG and FQ: funding acquisition. FQ and JO’D: supervision. FG and LC: visualization. FG, ZS, and MH: writing—original draft. All authors: writing—review and editing. All authors contributed to the article and approved the submitted version.

## Funding

The RFDS COVID-19 response was funded by the Commonwealth Government of Australia.

## Conflict of interest

The authors declare that the research was conducted in the absence of any commercial or financial relationships that could be construed as a potential conflict of interest.

## Publisher’s note

All claims expressed in this article are solely those of the authors and do not necessarily represent those of their affiliated organizations, or those of the publisher, the editors and the reviewers. Any product that may be evaluated in this article, or claim that may be made by its manufacturer, is not guaranteed or endorsed by the publisher.
